# Effects of Ambient Air Pollutants on Hospital Admissions among Children Due to Asthma and Wheezing-Associated Lower Respiratory Infections in Mysore, India: A Time Series Study

**DOI:** 10.3390/children10081322

**Published:** 2023-07-31

**Authors:** Sowmya Malamardi, Katrina Lambert, Jayaraj Biligere Siddaiah, Bircan Erbas, Padukudru Anand Mahesh

**Affiliations:** 1 Department of Public Health, School of Psychology & Public Health, College of Science Health and Engineering, La Trobe University, Melbourne, VIC 3083, Australia; dr.sonymalamardi@gmail.com (S.M.); k.lambert@latrobe.edu.au (K.L.); b.erbas@latrobe.edu.au (B.E.); 2 Department of Respiratory Medicine, JSS Academy of Higher Education & Research (JSSAHER), JSS Medical College, Mysore 570015, Karnataka, India; drjayarajbs@yahoo.com

**Keywords:** asthma, hospitalization, child, air pollutants, time series

## Abstract

Air pollutants are known to trigger asthma and wheezing-associated lower respiratory infections in children, but evidence regarding their effect on hospital admissions in India is limited. We conducted a time-series study over a period of five years to assess the role of ambient air pollutants in daily asthma-related hospital admissions in children in Mysore, India. Daily asthma and wheeze (associated with lower respiratory infections) admissions were modelled using a generalised additive model (GAM) to examine the non-linear effects and generalised linear models (GLM) for linear effects, if any. Models were adjusted by day of the week and lag days, with smooth terms for time, maximum temperature, and relative humidity, and they were stratified by sex and age group. Of the 362 children admitted, more than 50% were boys, and the mean age was 5.34 years (±4.66). The GAMs showed non-linear associations between NO_2_, PM_2.5_, and NH_3_. For example, a 10 µgm^−3^ (or 10%) increase in NO_2_ increased admissions by 2.42. These non-linear effects were more pronounced in boys. A linear effect was detected for PM_10_ with a relative risk (95% CI) of 1.028, 1.013, and 1.043 with admission. Further research is needed to explore whether these findings can be replicated in different cities in India. Air pollution needs to be controlled, and policies that focus on lower cut-off levels for vulnerable populations are necessary.

## 1. Introduction

Asthma is a non-communicable disease most common in children and adolescents [1]; it is characterised by coughing, wheezing, dyspnoea, and chest tightness [2], and requires immediate medical care at a hospital [3,4]. Asthma in children is an important global health burden [5]. Several environmental, individual, and familial factors are associated with hospitalisation for asthma in children [6,7,8,9,10,11]. Understanding the triggers for hospitalization is important, as a high rate of admissions is a burden on the individual, the carer, and the hospital system.

In India, air pollution is one of the leading environmental risk factors contributing to the global burden of disease (GBD) [12], especially in urban areas [11]. Key pollutants are particulate matter of an aerodynamic diameter no greater than 2.5 µm or 10 µm (PM_2.5_, PM_10_), nitrogen dioxide (NO_2_), or sulphur dioxide (SO_2_) [13,14]. In India, both burning of biomass and industrial activity (fertilisers) are common, and thus studying NH_3_ emissions is also important [15]. However, studies exploring the effects of air pollution on hospital admissions for asthma are limited to major cities in India, such as Delhi [16,17,18,19,20]. Hence, it is important to understand the impacts of air pollution on other cities with differing levels of environmental factors and that are not heavily polluted, such as Mysore, India.

Hospital admissions related to asthma are adverse events due to constriction of the airways and inflammation from exposure to air pollutants [21], which critically increase risk of hospitalisation [22]. The condition is episodic and reversible based on severity and individual response, but it is also preventable [23,24]. Emergency departmental visits due to asthma are high in children and adolescents [25], contributing to a higher disease burden for children, families, and hospitals. We aimed to study the association between air pollution and daily asthma-related hospital admissions (including wheeze-associated lower respiratory infections) in children and adolescents. A secondary aim was to assess differences in estimate effects based on sex and age strata.

## 2. Materials and Methods

We conducted a retrospective time-series study from 1 January 2017 to 31 December 2021. The study population comprised children aged less than 9 years and adolescents aged 10–19 years with asthma and wheezing-associated lower respiratory infections (WALRI) residing in Mysore, India (an area of 152.05 km^2^). Mysore is geographically situated in the southern part of the state of Karnataka, India, and lies at 12°18′ North latitude and 76°38′ East longitude, including ~4.17 million people. The study commenced after ethical approval was obtained from JSS Medical College and Hospital (ECR/387/Inst/KA/2013/RR-19). The data on asthma and WALRI hospital admissions on children and adolescents were composed of ICD-9 codes (493) and ICD-10 codes (J45, J46, or R06.2). A manual medical record abstraction by the medical staff and electronic health records review was conducted to collect data from the tertiary hospital. We included WALRIs as admissions in children 0–2 years of age, as asthma diagnosis is unreliable in this age group; this included all patterns of wheezing (never, transient early, late-onset, and persistent) [26,27]. Patients with unspecified asthma (J45.90x) and other associated severe respiratory infections were excluded. We reviewed the hospital records and harmonised the data from the daily hospital admissions. Patients admitted for any other cause were excluded. The counts of daily admissions for asthma exacerbations were noted. The daily counts of admissions did not comprise emergency room visits, as the treatment and management was different in the tertiary hospital. If the patient failed to respond to emergency care within 24 h, inpatient admission followed, and we have included those cases. Hence, we analysed these data as a continuous time-series study by achieving a sufficient sample size for reliable estimates, though the annual rate of admissions varied.

We included air pollutants particulate matter of up to 2.5 µm in diameter (PM_2.5_) and up to 10 µm (PM_10_), sulphur dioxide (SO_2_,), and nitrous oxide (NO_2_). We also included NH_3_, as ammoniated aerosols form a part of the particulate matter [28]. The air quality monitoring stations set up by India’s National Ambient air quality Monitoring Program (NAMP) were our sources of ambient air pollutants data collected from 1 January 2017 to 31 December 2021 [29]. In the city of Mysore, a manual air quality monitoring station was set up twice a week, which collected data on air pollutant parameters for 24 h, totalling 104 observations in each year. The protocol for data collection was at least two measurements per week, but the day of measurement varied depending on the period of data collection. Each pollutant was measured twice a week. We took a running cumulative average in between the two measures for each day of week. We also adjusted for these lags in our models. For example, if PM_2.5_ was collected only on 6 January 2017 and 10 January 2017, we took the measurement on the 6th, averaged across the 4 days before the next measurement, and then followed this process at the next measurement.

Data on daily measure of weather parameters, including maximum and minimum temperature (in degree Celsius) and percentage of relative humidity, were obtained from the appropriate publicly accessible databases www.weatheronline.in (accessed on 11 May 2022) and national ambient air quality monitoring station (NAAQS). Individual characteristics such as weight/height at admission, sociodemographic characteristics, and family history of asthma were obtained from the hospital records. A standard and widely accepted revised Kuppaswamy scale (2021) was used to capture socio-economic status (SES) data [30]. The CDC BMI classification designed to monitor the growth of children and teens aged 2–19 years was used in the study and standardised for the study population [31].

We conducted a time-series analysis of daily hospitalisations as the outcome variable and each pollutant’s daily average concentrations as the primary exposure variables separately. We assessed correlations between air pollutants (PM_2.5_, PM_10_, NO_2_, SO_2_, and NH_3_) and the meteorological factors (maximum temperature, relative humidity, and precipitation), thus generating a correlation matrix using Spearman’s correlation statistics. We described the characteristics of each admission and used statistical tests for comparisons depending on the distribution and definition of each variable. All data were analysed using Stata IC16 (STATA Corp., College Station, TX, USA).

We used Generalised Additive Models (GAMs) to assess the associations between each pollutant and admissions. As daily hospital admissions follow a probability of an event, we used Poisson distribution with the log link. Over dispersion was not present. SO_2_ was below detectable levels during the study and we did not include it in subsequent analyses. We first fitted a (GAM) to assess any non-linear effects for each air pollutant while adjusting for weather parameters (temperature and humidity), day of the week, and season. We chose the best-fitting models, which yielded the minimum Akaike information criterion (AIC) and maximum log links. If the GAMs showed no statistically significant linear effects, we reverted to generalised linear regression models (GLMs). R software (4.2.2v) was used for the analysis, and the results were reported as effect measures (RRs) and 95% confidence intervals (CI). The *p*-values less than 0.05 were considered statistically significant. The graph plotting for this time series study involving R software (4.2.2v) used mgcv, mgcViz, and ggplot2 packages [32,33].

## 3. Results

There were 362 admissions between 2017 and 2021, with a total of 194 young (aged less than five years) and 168 older (aged more than or equal to five years) patients admitted to the tertiary care hospital in Mysore during the study period (Table 1). The participants’ mean age was 5.34 years (±4.66), with 83.89% aged less than ten years and 65.56% boys (*n* = 232). The mean (SD) average duration of hospital stay was slightly higher among boys, 3.77 (3.10) days, compared to girls, 3.54 (1.88). Of the total sample, 11.84% belonged to a lower socioeconomic status. Most of the children had a normal BMI (79.4%), followed by obese (10.3%), underweight (6.4%) and overweight 15 (4.2%). We observed a family history of asthma in a small number of subjects: paternal (11.71%), maternal (7.43%) and siblings (2.61%).

Over the 1826-day duration of the study, there were a total of 362 hospital admissions for asthma (N = 211) and WALRI (N = 151) (Table 2). We display the time series graph in Supplementary Figure S1. There was a decline in admissions during the COVID-19 lockdown in India. The admissions peaked during the monsoon (43.89%), then winter (21.39%), autumn (18.61%) and summer (16.11%). No difference was observed between boys and girls. There was a significant difference in admissions across months (*p* < 0.0001), and September had the highest admissions in a year, followed by August (Table 2). The number of asthma admissions on weekdays was higher than at weekends, and the highest numbers reported were on Tuesdays and Thursdays (*p* < 0.012). Small to moderate correlations were observed between air pollutants and weather parameters in this study (Supplementary Table S1).

We examined each air pollutant separately to assess the potential non-linear effects on daily asthma admission using the GAM framework. The effects of NO_2_, PM_2.3_, and NH_3_ were non-linear in adjusted models (Table 3). PM_10_ was not associated with admissions related to asthma/WALRI (Table 3). When models were stratified by boys/girls, our models showed that daily admissions in boys were higher with an increase in the air pollutants NO_2_, PM_2.5_, and PM_10_, but the same was not true for girls. We constructed smooth plots to show the non-linear effects between the pollutants and outcome (online Supplementary Figures S2–S5). When the results were stratified for boys, we found that the fitted non-linear curve for NO_2_ and PM_2.5_ resembled the figure for all children, suggesting that boys generally drive this trend. Interestingly, PM_10_ exposures only among boys were non-linear (online Supplementary Figure S4) but the same did not apply to girls. Among children, the effect of NH_3_ was non-linear and at higher levels of NH_3_ (>20 µgm^−3^), the number of hospital admissions declined (online Supplementary Figure S5).

The log relative risk of asthma hospital admissions for the PM_10_ effects showed linear relationships (Table 4). Daily exposure to PM_10_ was associated with daily admissions RR = 1.028 (95% CI 1.013, 1.043); there were no significant differences across sex and age groups.

## 4. Discussion

The current study is the first conducted in Mysore, India, to show that ambient air pollutants increase the risk of hospital admissions for asthma and wheeze (WALRI) among children and adolescents. Generative Additive Models detected non-linear associations with NO_2_, PM_2.5_, and NH_3_. The expected risk of daily asthma admissions, given children exposed to PM_10_, was linear with a RR = 1.028 and 95% CI (1.013, 1.043). These effects were pronounced in boys, but no such associations were observed among girls.

Our GAM models show that an increase in NO_2_ concentration was associated with an increase in asthma-related hospital admissions at lower levels, with a similar association having been observed in studies in the Netherlands [34] and the US [35]. Traffic pollutants contribute NO_2_ in the atmosphere, which is strongly associated with vehicular emissions and secondary formation of secondary pollutants (such as fine particles, ozone, and others) [36]. Recent studies reporting emissions from buses [37] have shown a high contribution of ambient NOx, especially in cities like Mysore [38].

PM_2.5_ had a non-linear association with asthma hospitalisation in our study, with increasing levels above 10 µgm^−3^ associated with increased daily asthma hospital admission rates. Similarly, in a study conducted in Ahmedabad, an increase in the concentration of PM_2.5_ was associated with an increase in the respiratory admissions (winter: RR = 1.16 95% CI = 1.007–1.23) [39]. With the interquartile range increase in PM_2.5_, a Taiwan study observed significant increases in asthma hospitalisation among children under 18 years old (RR = 1.156; CI = 1.142–1.170; *p* < 0.001) [24]. PM is a complex mixture of solid and liquid particles suspended in air and originates from the combustion process of diesel and gasoline-powered vehicles, burning of biomass, and burning of coal to generate power [24], affecting various pathways and causing differential health effects [40,41]. PM_2.5_ penetrates alveolar portions of the lung; the particles translocate to blood circulation through these alveolar capillaries while PM_10_ is trapped in the upper part of the lower respiratory tract, thus causing increased dose responses effects more closely related to PM_2.5_ than PM_10_ [42]. However, PM_2.5_ related to residential biomass fuel emissions is the most significant contributor to ambient levels across India, followed by the combustion of agricultural residue [43]. A study has shown that an increase in PM_2.5_ due to vehicular emissions over the years comprised 50% of vehicular emissions from heavy diesel vehicles and PM_10_ of all types of emissions [44].

In our study, the effect of PM_10_ on asthma-related hospital admissions was linearly associated. Samoli et al. showed a 2.54% increase in the number of daily paediatric asthma hospital admissions with increased 10 µgm^−3^ PM_10_ exposure [45]. With an interquartile range increase in PM_10_, a Taiwan study observed significant increases in asthma hospitalisation (RR = 1.120; CI = 1.107–1.134; *p* < 0.001) [24]. For India, residential and industrial sectors were the largest contributors, followed by power and transportation [46], while non-exhaust emission sources can also play a significant role in PM_10_ emissions [19].

We also identified some effects of NH_3_ exposure. There are few studies related to NH_3_ and asthma hospitalisation, but some studies have evidenced the effects [47,48]. Sources of NH_3_ include agriculture processes, industries (fertiliser and pharmaceutical) and vehicular emissions [48]. The effects of NH_3_ may be detrimental, as studies have shown that children exposed to such environments experience asthma and other respiratory symptoms [49,50]. In 2017, more than half India’s population (56%) remained exposed to household air pollution, and about one-third of the population still uses solid fuels in Karnataka.

Chakraborty (2014) in Kolkata, India, studied the effects of PM_10_ using GAM regression analysis and found that PM_10_ was associated with increased emergency asthma hospitalisation in school-aged children [51]. However, our study is on admissions and is not directly comparable. However, many of the studies in India focus on emergency department (ED) presentations, and a portion of these would account for admissions themselves, so they are relevant in terms of which pollutants may be impacting ED [20] and respiratory admissions [39]. The scatter plots present smooth S-shaped curves (Supplement Figures S3 and S4), where the risk of admission increases from a low level below a critical exposure concentration threshold to a high level above it; the curves appear to be smooth, meaning associations are linear at low doses at low levels [52,53,54]. Similarly, at low concentration levels of PM_2.5_, there is a short-term risk associated with exacerbation of asthma and associated hospital admissions. We observed exposure concentration response through our time series epidemiological study, presenting the linear and non-linear association of PM_10_, PM_2.5_, NO_2_, and NH_3_ and asthma-related hospital admissions in children. But to understand how a change in exposure would change the risks associated with respiratory health, a higher modelling, such as the use of Bayesian networks and dynamic simulation models, potentially supports in understanding the causally interpretable non-linear pattern of association [55].

Physiologically, the airways of boys are smaller than those of girls [56,57,58,59], making them prone to an increased number of respiratory infections and prevalence of asthma [60]; thus, they are twice as likely to be hospitalised compared to girls. Our study showed a greater number of asthma admissions among boys (*n* = 65.56%), with higher admissions for some pollutants in boys and not girls. Many studies have shown that air pollution affects boys and not girls. A study in Taiwan observed high asthma admissions among boys compared to girls, which is consistent with our findings [24]. An extensive database study in the US observed similar gender differences [61].

The viral respiratory panel (VRP) test is designed to detect viral pathogens in the respiratory tract, which may contribute to the exacerbation of chronic obstructive pulmonary disease (COPD), as the upper and lower respiratory tract infections are caused by a broad range of microbes and not only bacteria [62]. Opara et al. have discussed the advantages of VRP tests in acute exacerbation of COPD (AECOPD) among 18–65-year-old adults. Studies have also demonstrated the benefits of utilising the rapid panel test of respiratory viral and atypical bacteria on the aetiological diagnosis of acute lower respiratory infection (ALRI) and rational use of antibiotics [63]. A study conducted in the UAE reported that RVP multiplex polymerase chain reaction (PCR) tests in children offer benefits such as lower hospitalisation rates and rational use of antibiotics, making them a cost-effective clinical management tool, and may be beneficial due to the viral aetiology in asthma [64]. Unfortunately, VRP was not tested in children admitted with lower respiratory tract infections in tertiary centres in India.

Overall, this study had several strengths. The relationship between pollutants and asthma admissions was evaluated over five years, which adds robustness to the observations. A major strength of our study is that we had access to hospital records for a major hospital in Mysore, which is used by most of the population, and can be generalisable to populations like those in Mysore. Most of the studies in India have been conducted in cities with higher air pollution than Mysore. This study was the first in South India to focus on children; it evaluated the relationship between air pollutants and asthma-related conditions in a city with moderate to low pollution levels and provided insight into the impact of air pollution on asthma admissions in regions with lower pollution levels. The study had several limitations as well. A small sample size (in this case, 0 to 1 admissions on a given day) limits statistical power when conducting stratified analyses. Another limitation is the combination of asthma and WALRI in a single group. The exposure data in the first half of the study did not include daily measurements, so we could not assess lags. Ozone is also an important air pollutant. However, data collection and analysis occurred after this study began, and the exclusion of ozone may influence the findings. Our study observed a slight decline in hospital admissions after the first 3 years. Hospitalisation rates could reflect decreases in the frequency and severity of asthma exacerbations. However, other factors should also be considered, such as the COVID-19 epidemic that affected the dependent (asthma admissions) and exposure factors (ambient air pollutants); further research on post-COVID-19 trends would provide further insight. Unlike in prospective cohort studies, the multipollutant model interpretation may not strongly add to the casual interpretation. Therefore, we have opted not to conduct multi-pollutant models, with single pollutant models being the main interpretation from the analysis.

## 5. Conclusions

Even though some of the pollutants are slightly above the WHO standard, these findings need to be considered when government departments are revisiting the development of pollutant standards. India is focused on self-committed targets for reducing emissions, in accordance with the Paris Agreement [12]. Our study provides further insights into this growing problem of environmental triggers influencing respiratory-related conditions in children and adolescents. Further research over longer periods and different cities in India is necessary to replicate our findings.

## Figures and Tables

**Table 1:** Descriptive statistics.

Variable	All	Boys	Girls	*p* Value
N (%)	362	232 (64.08)	130 (35.91)	
Mean age ± SD	5.34 (±4.66)	5.69 (4.85)	5.16 (4.47)	
Age group, N (%)				
<5 years (N = 194)	194, (53.59)	116 (59.79)	62 (31.95)	
≥5 years (N = 161 + 7)	168 (46.40)	116 (69.04)	68 (40.47)	
Diagnosis N (%)				
Asthma	211 (57.34)	134 (57.58)	77 (56.91)	
WALRI	151 (42.66)	98 (42.42)	53 (43.09)	
Weight (in kgs), mean (SD)	18.07055 (12.5943)	17.61 (12.04156)	18.94906 (13.59612)	
Height (in cm), mean (SD)	103.2295 (28.22597)	102.2735 (27.19837)	105.0516 (30.12417)	
BMI, mean (±SD)	15.48876 (3.30534)	15.48056 (3.408503)	15.50438 (3.113445)	*p* = 0.511
BMI (min, max)	(9.002058, 27.6601)	(10.38781, 27.6601)	(9.002058, 27.38558)	
BMI group N (%)				
Underweight	23 (6.4)	15 (6.4)	23 (6.4)	*p* = 0.70
Normal	285 (79.4)	189 (80.1)	285 (79.2)	
Overweight	15 (4.2)	7 (3.0)	15 (4.2)	
Obese	37 (10.3)	25 (10.6)	37 (10.3)	
Average hospital stays (in days), mean (SD) (>3 days)	3.69 (2.74)	3.77 (3.10)	3.54 (1.88)	
Total duration of hospital stays (in days) N (min, max)	360 (0, 28)	236 (0, 28)	124 (0, 10)	
No of previous hospital admissions N (min, max)	208 (1, 12)	137 (1, 12)	71 (1, 7)	
No of previous hospital admissions within 12 months N (min, max)	121 (1, 12)	82 (1, 4)	39 (1, 12)	
No of previous hospital admissions within 28 days N (min, max)	37 (1, 4)	24 (1, 2)	13 (1, 4)	
Socioeconomic status N (%)				
Lower	36 (11.84)	27 (13.30)	9 (8.91)	
Lower middle	76 (25.00)	46 (22.66)	30 (29.70)	
Middle	167 (54.93)	117 (57.64)	50 (49.50)	
Upper middle	25 (8.22)	13 (6.40)	12 (11.88)	
Family history of Asthma N (%)				
Paternal history N (%)	41 (11.71)	18 (7.86)	23 (19.01)	
Maternal history N (%)	26 (7.43)	15 (6.55)	11 (9.09)	
Sibling history N (%)	7 (2.61)	4 (2.31)	3 (3.16)	
Season N (%)				
Winter	77 (21.39)	44 (18.64)	33 (26.61)	
Summer	58 (16.11)	38 (16.10)	20 (16.13)	
Monsoon	158 (43.89)	112 (47.46)	46 (37.10)	
Autumn	67 (18.61)	42 (17.80)	25 (20.16)	

Note: N = Frequency/number; % means percentage; SD: standard deviation; mean = average; min: minimum; max: maximum; total = total number of hospital admissions from 1 January 2017 to 31 December 2021.

**Table 2:** Summarising descriptive variables (mean of air pollution levels and meteorological variables, hospital admissions in Mysore, 2017–2021).

Variable	Mean (SD)	Percentile				Min	Max
		25	50	75	90		
PM_2.5_ (µgm^−3^)	25.68 (5.35)	24.00	26.40	29.00	31.00	8.00	41.40
PM_10_ (µgm^−3^)	50.37 (8.61)	46.00	51.00	55.00	59.00	20.00	77.00
NO_2_ (µgm^−3^)	15.72 (2.55)	14.09	15.10	16.62	19.70	11.20	24.20
NH_3_ (µgm^−3^)	14.42 (2.98)	12.69	14.11	15.64	17.03	9.62	38.30
Temperature (max) in Celsius	30.91 (2.34)	29.00	31.00	32.00	34.00	21.00	38.00
Humidity (%)	73 (26.02)	53	76.00	65.2	86.3	17.0	98.4
Asthma admission (total 362)	0.1982 (0.548)	0	0	0	1	0	4
Weekdays *	N (%)						
Monday	51 (14.09)	0	0	0	1	0	3
Tuesday	61 (16.85)	0	0	0	1	0	3
Wednesday	49 (13.54)	0	0	0	1	0	3
Thursday	59 (16.30)	0	0	0	1	0	4
Friday	58 (16.02)	0	0	0	1	0	4
Saturday	44 (12.15)	0	0	0	1	0	3
Sunday	40 (11.05)	0	0	0	1	0	4
Total	362 (100)	0	0	0	1	0	4
Month **	N (%)						
January	31 (8.56)	0	0	0	1	0	3
February	20 (5.52)	0	0	0	1	0	2
March	25 (6.91)	0	0	0	1	0	2
April	16 (4.42)	0	0	0	0	0	3
May	08 (2.21)	0	0	0	0	0	2
June	17 (4.70)	0	0	0	0	0	3
July	34 (9.39)	0	0	0	1	0	4
August	53 (14.64)	0	0	0	1	0	3
September	60 (16.57)	0	0	1	1.5	0	4
October	52 (14.36)	0	0	0	1	0	4
November	25 (6.91)	0	0	0	1	0	3
December	21 (5.80)	0	0	0	1	0	3
Total	362 (100)	0	0	0	1	0	4

Note: SD: standard deviation; min: minimum; P25: 25th percentile; P75: 75th percentile; Min: minimum; Max: maximum; total = total number of hospital admissions for period 1 January 2017 to 31 December 2021. * *p* < 0.05, ** *p* < 0.001.

**Table 3:** Summary of the GAMs for daily asthma admissions.

GAM *	All Participants * (N = 362)	Boys * (N = 232)	Girls * (N = 130)	Children Less than 5 Years * (N = 194)	Children More than Equal to 5 Years * (N = 168)
Air Pollutants + s (Time) + Day of Week Categorical + s (Tempmax) + s (Relative Humidity)	Edf (*p* Value); logLIK	Edf (*p* Value); logLIK	Edf (*p* Value); logLIK	Edf (*p* Value); logLIK	Edf (*p* Value); logLIK
NO_2_	2.428 (0.0223); −879.839	2.483 (0.00814); −651.784	1.000 (0.931); −109.829	1.000 (0.931); −179.266	1.000 (0.740); −145.630
PM_2.5_	3.910 (0.0113); −876.890	3.844 (0.016); −651.283	1.000 (0.572); −109.67	1.000 (0.790); −179.235	1.000 (0.762); −145.640
PM_10_	1.006 (0.0534); −884.725	2.049 (0.0271); −655.08	1.000 (0.916); −109.828	1.000 (0.959); −179.269	1.000 (0.640); −145.575
NH_3_	4.810 (<0.0001); −831.068	4.532 (<0.0001); −631.238	5.705 (0.00074); −357.64	3.751 (<0.0001); −553.186	4.919 (<0.0001); −466.177

* All estimates adjusted for lag days, weekdays, maximum temperature, and relative humidity. Note: Each of the study variables was used in the generalised additive models (GAMs) with the Poisson link to explore the association between the daily asthma hospitalisation and air pollutants concentration (in µgm^−3^) as the primary exposure variable. In the GAM analysis, columns represent the estimates of GAM model for daily asthma hospitalisation in children and air pollutant concentrations adjusted for the day of the week, lag days, maximum temperature in degree Fahrenheit, and relative humidity in percentages. Smoothing parameters were time, maximum temperature, and relative humidity. The continuous variables represent PM_10_—particulate matter of diameter 10 µm or less; PM_2.5_—particulate matter of diameter 2.5 µm or less; NO_2_—nitrogen dioxide; NH_3_—Ammonia; and SO_2_—sulphur dioxide as µgm^−3^; and daily asthma admission as counts. The estimates are presented for all participants (children), boys, girls, children less than 5 years old, and children more than or equal to 5 years old from column 2 to 6. The *p*-values are reported for each of the estimates and the logLINK exponentiates the linear predictors where the higher value presents a better fit.

**Table 4:** Summary estimates of GLM for daily asthma admissions.

GLM	All Participants * (N = 362)	Boys * (N = 232)	Girls * (N = 130)	Children Less than 5 Years * (N = 194)	Children More than Equal to 5 Years * (N = 168)
Air Pollutants	RR (95% CI); *p* Value; logLIK	RR (95% CI); *p* Value; logLIK	RR (95% CI); *p* Value; logLIK	RR (95% CI); *p* Value; logLIK	RR (95% CI); *p* Value; logLIK
NO_2_	Non-linear (Refer to Figure S2)	Non-linear	0.995 (0.915, 1.082); 0.671; −109.91	0.956 (0.878, 1.042); 0.314; −135.56	1.005 (0.912, 1.108); 0.905; −146.23
PM_2.5_	Non-linear (Refer to Figure S3)	Non-linear	0.994 (0.971, 1.017); 0.642; −109.810	0.993 (0.969, 1.018); 0.613; −135.96	0.990 (0.961, 1.019); 0.509; −146.03
PM_10_	1.028 (1.013, 1.043); **<0.0001**; −104.634	Non-linear (Refer to Figure S4)	0.998 (0.977, 1.020); 0.914; −109.910	0.999 (0.970, 1.029); 0.988; −136.08	0.987 (0.952, 1.024); 0.507; −146.02
NH_3_	Non-linear (Refer to Figure S5)	Non-linear	Non-linear	Non-linear	Non-linear

* Adjusted for lag days, maximum temperature, and relative humidity. Note: Each of the study variables was used in the generalised linear models (GLMs) with the Poisson link to explore the association between the daily asthma hospitalisation and air pollutants concentration (in µgm^−3^) as the primary exposure variable. In the GLM analysis, columns represent the estimates of GAM model for daily asthma hospitalisation in children and air pollutant concentrations adjusted for the lag days, maximum temperature in degree Fahrenheit, and relative humidity in percentage. The continuous variables represent PM_10_—particulate matter of diameter 10µm or less; PM_2.5_—particulate matter of diameter 2.5 µm or less; NO_2_—nitrogen dioxide; NH_3_—ammonia; and SO_2_—sulphur dioxide as µgm^−3^; and daily asthma admission as counts. The estimates are presented for all participants (children), boys, girls, children less than 5 years old, and children more than or equal to 5 years from column 2 to 6 as RR (Relative Risk). The *p* values are reported for each of the estimates and the logLIK exponentiates the linear predictors where the higher value present a better fit and *p*-value < 0.0001 is bold.

## Data Availability

The data and materials used in this study can be made available on request through an email to the corresponding author of this study. The analysis code is obtainable from the corresponding author upon reasonable request.

## Supplementary Materials

The following supporting information can be downloaded at: https://www.mdpi.com/article/10.3390/children10081322/s1, Figure S1: Time series graphs of all daily asthma hospital admissions for the year 2017–2021; Figure S2: GAM plot* of NO_2_ for children (*n* = 362); Figure S3: GAM plot* of PM_2.5_ for children (*n* = 362); Figure S4: GAM plot* of PM_10_ for boys (*n* = 232); Figure S5: GAM plot* of NH_3_ for children (*n* = 362); and Table S1: Spearman’s correlations between air pollutants and weather parameters.
