# Supplementary material for: Effects of Ambient Air Pollutants on Hospital Admissions among Children Due to Asthma and Wheezing-Associated Lower Respiratory Infections in Mysore, India: A Time Series Study
Source: Children (Basel). 2023 Jul 31;10(8):1322. doi: 10.3390/children10081322 (PMC10453753; doi:10.3390/children10081322)
Supplement: Supplementary file 1 [file children-10-01322-s001.zip › children-2478852-supplementary.pdf]

Table S1. Spearman’s correlations between air pollutants and weather parameters

Correlation Matrix

		**PM_{10}**	**$PM_{2.5}$**	**NO_2**	**NH_3**	**SO_2**	**Temp-max**	**Humidi-ty**	**Precipi-tate**
PM_{10}	Spearman's rho	—							
	p-value	—							
$PM_{2.5}$	Spearman's rho	0.618 ***	—						
	p-value	< .001	—						
NO_2	Spearman's rho	0.543 ***	0.402 ***	—					
	p-value	< .001	< .001	—					
NH_3	Spearman's rho	0.253 ***	0.122 ***	0.361 ***	—				
	p-value	< .001	< .001	< .001	—				
SO_2	Spearman's rho	-0.037	-0.009	0.101 ***	0.085 ***	—			
	p-value	0.118	0.709	< .001	< .001	—			
Tempmax	Spearman's rho	0.206 ***	0.106 ***	0.034	0.213 ***	-0.075 **	—		
	p-value	< .001	< .001	0.150	< .001	0.001	—		
Humidity	Spearman's rho	-0.339 ***	-0.292 ***	-0.118 ***	-0.156 ***	0.044	-0.719 ***	—	
	p-value	< .001	< .001	< .001	< .001	0.058	< .001	—	
Precipita-tion	Spearman's rho	0.145 ***	0.098 ***	0.232 ***	-0.149 ***	-0.056 *	-0.215 ***	0.292 ***	—
	p-value	< .001	< .001	< .001	< .001	0.017	< .001	< .001	—

Note. * $p < .05$, ** $p < .01$, *** $p < .001$

Data describes the Correlation matrix for the air pollutants and the weather parameters. The corresponding p values are reported below each correlation co-efficient values. Significant p values that is $p<0.05$ are made bold. The variables are continuous represent PM_{10}, particulate matter of diameter 10μm or less; $PM_{2.5}$, particulate matter of diameter 2.5μm or less; NO_2, nitrogen dioxide; NH_3, Ammonia; and SO_2, sulfur dioxide as μgm^{-3}; maximum temperature in degree Celsius; Relative Humidity in percentage; Precipitation in percentage.

FIGURE LIST

Supplement Figure S1 Time series graphs of all daily asthma hospital admissions for the year 2017-2021

Supplement Figure S2 is GAM plot* of NO_2 for children (n=362).

Supplement Figure S3 GAM plot* of $PM_{2.5}$ for children (n=362)

Supplement Figure S4 GAM plot* of PM_{10} for boys (n=232)

Supplement Figure S5 GAM plot* of NH_3 for children (n=362)

FIGURE (TITLES &LEGENDS)

Supplement Figure 1 Time series graphs of all daily asthma hospital admissions for the year 2017-2021

Data presented for the daily asthma hospital admissions in children (n=362) over the period of five years (2017-2021). In the figure, the x-axis constitutes the time period (labelled half yearly) and the y axis reports the daily count in the hospital admissions due to asthma and wheezing associated lower respiratory infections (WALRI) in children and adolescents represented as navy blue lines in the plot.

Supplement Figure 2 is GAM plot* of NO_2 for children (n=362).

A multivariable smooth scatter plot is presented for estimation of the smooth (number of admissions) for predictor (NO_2) done by back fitting. Smooth line in solid red colour show the fitted equation and the dashed lines in blue are the 95% Confidence intervals. Circles represent the individual observations residues. The x axis represents the concentration of the air pollutant NO_2 in μgm^{-3} and the y axis represented the rate of asthma hospital admission with smoothing among children (n=362). Other factors lag days, day of the week, maximum temperature, and humidity were adjusted in the model for time series analysis.

Supplement Figure 3 GAM plot* of $PM_{2.5}$ for children (n=362)

A multivariable smooth scatter plot is presented for estimation of the smooth (number of admissions) for predictor ($PM_{2.5}$) done by back fitting. Smooth line in solid red colour show the fitted equation and the dashed lines in blue are the 95% Confidence intervals. Circles represent the individual observations residues. The x axis represents the concentration of the air pollutant $PM_{2.5}$ in μgm^{-3} and the y axis represented the rate of asthma hospital admission with smoothing among children (n=362). Other factors lag days, day of the week, maximum temperature, and humidity were adjusted in the model for time series analysis.

Supplement Figure 4 GAM plot* of PM_{10} for boys (n=232)

A multivariable smooth scatter plot is presented for estimation of the smooth (number of admissions) for predictor (PM_{10}) done by back fitting. Smooth line in solid red colour show the fitted equation and the dashed lines in blue are the 95% Confidence intervals. Circles represent the individual observations residues. The x axis represents the concentration of the air pollutant PM_{10} in μgm^{-3} and the y axis represented the rate of asthma hospital admission with smoothing among boys (n=232). Other factors lag days, day of the week, maximum temperature, and humidity were adjusted in the model for time series analysis.

Supplement Figure 5 GAM plot* of NH_3 for children (n=362)

A multivariable smooth scatter plot is presented for estimation of the smooth (number of admissions) for predictor (NH_3) done by back fitting. Smooth line in solid red colour show the fitted equation and the dashed lines in blue are the 95% Confidence intervals. Circles represent the individual observations residues. The x axis represents the concentration of the air pollutant NH_3 in μgm^{-3} and the y axis represented the rate of asthma hospital admission with smoothing among children (n=362). Other factors lag days, day of the week, maximum temperature, and humidity were adjusted in the model for time series analysis.

Supplement Figure S1: Time series graphs of all daily asthma hospital admissions for the year 2017-2021

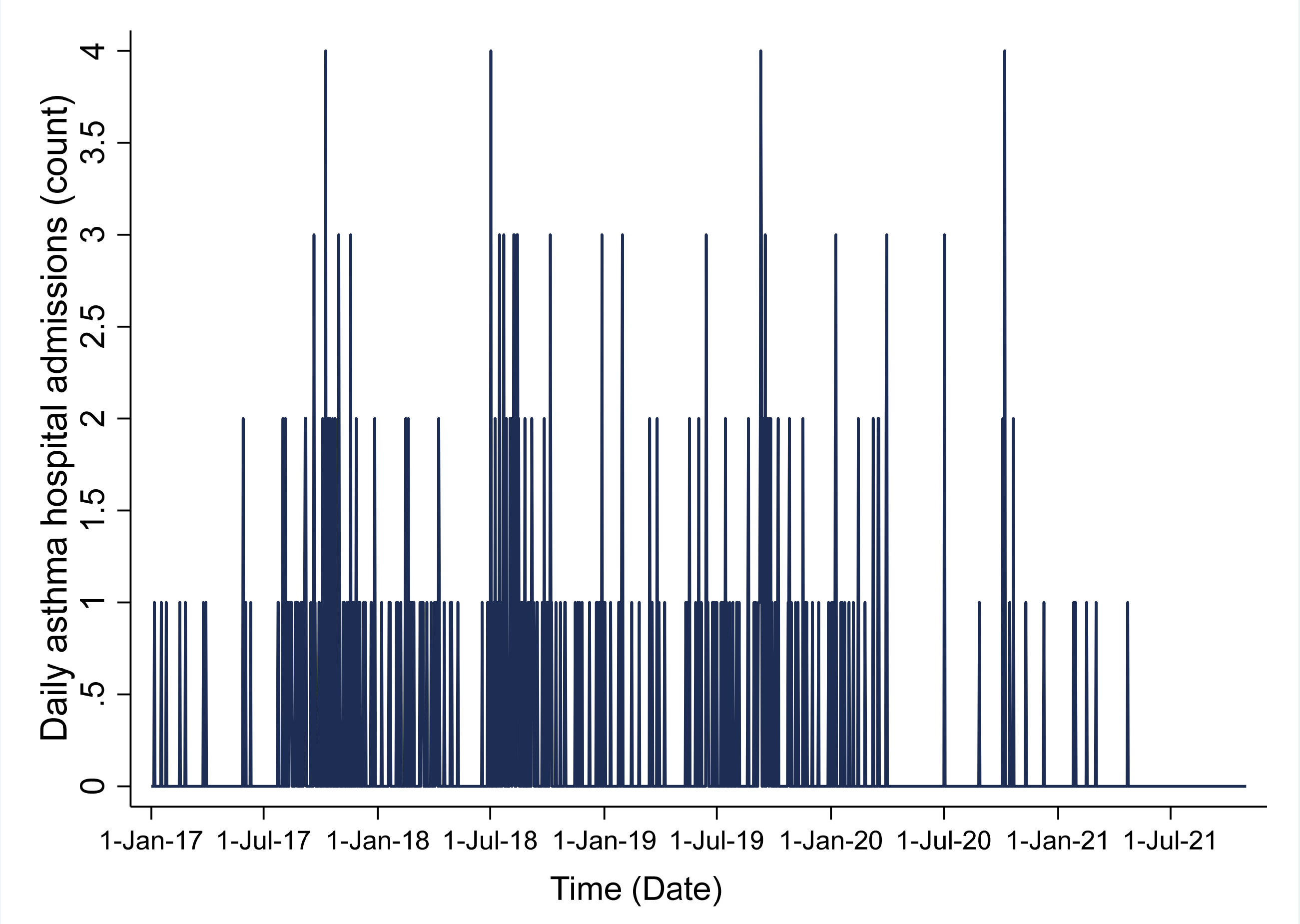

Supplement Figure S2 is GAM plot* of NO_2 for children (n=362).

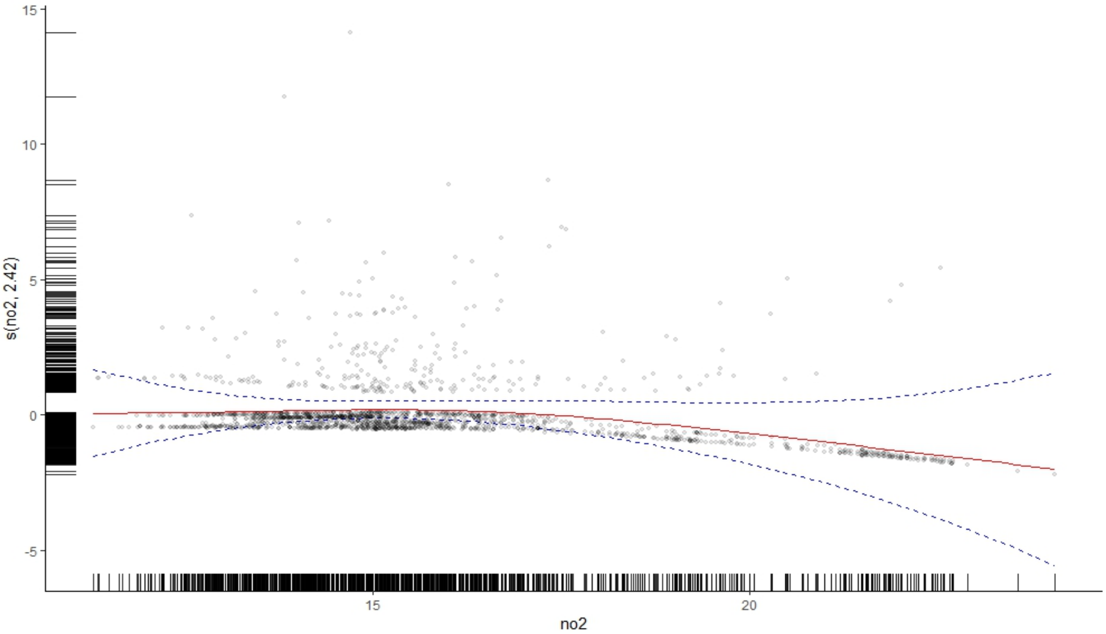

*Adjusted for the lag days, time, maximum temperature, and humidity

Supplement Figure S3 GAM plot* of $PM_{2.5}$ for children (n=362)

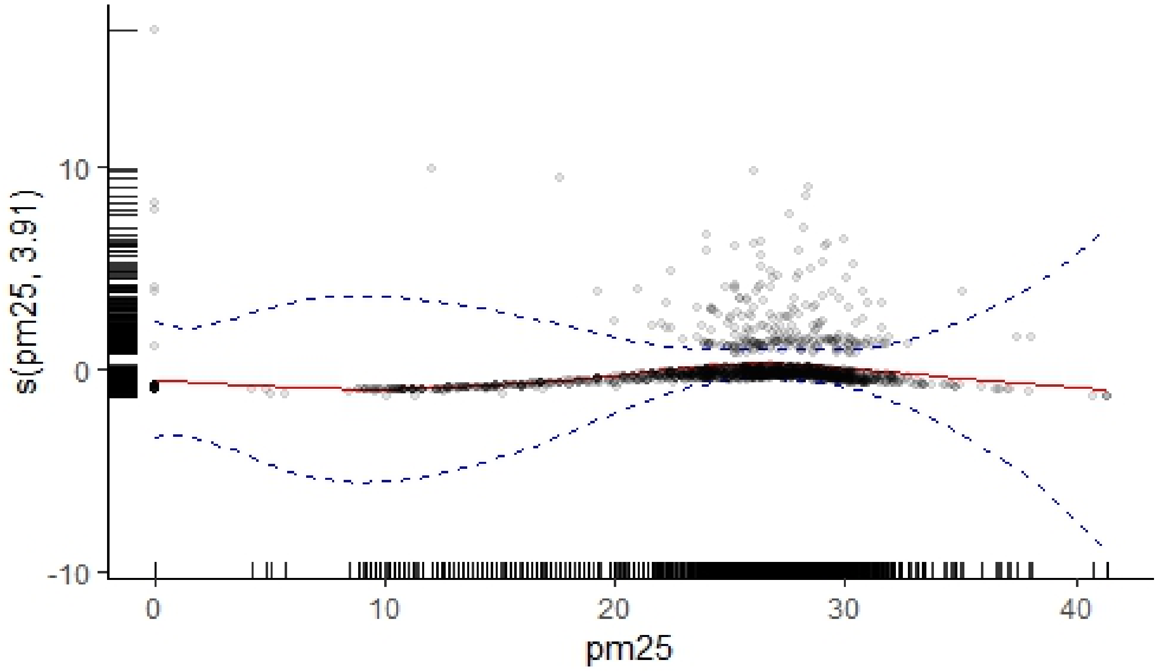

*Adjusted for the lag days, time, maximum temperature and humidity

Supplement Figure S4 GAM plot* of PM_{10} for boys (n=232)

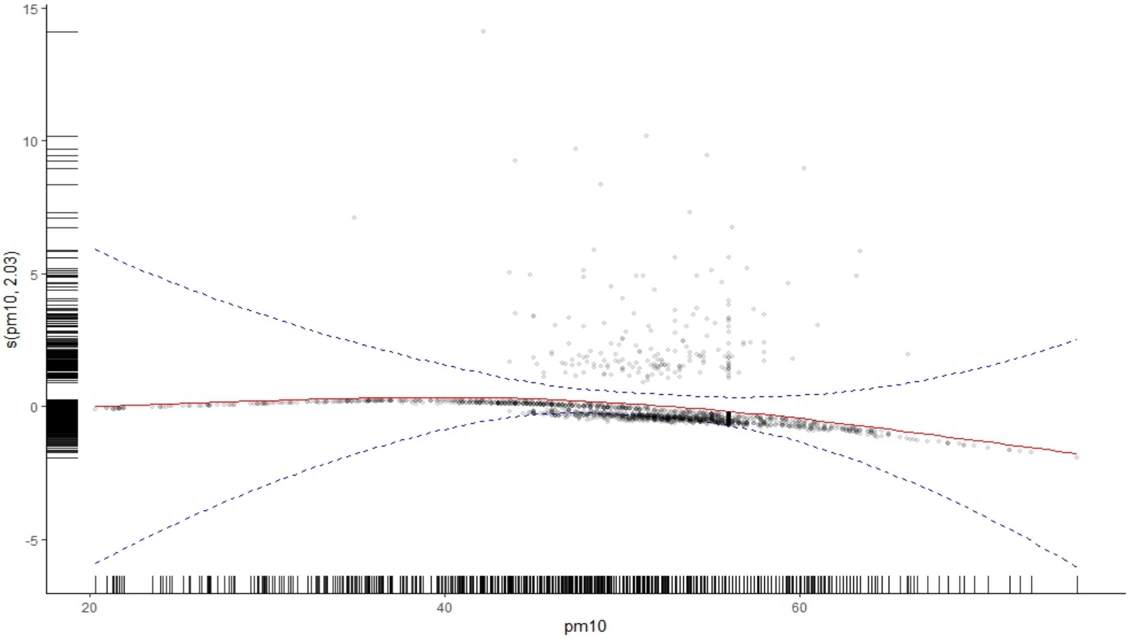

*Adjusted for the lag days, time, maximum temperature, and humidity

Supplement Figure S5 GAM plot* of NH_3 for children (n=362)

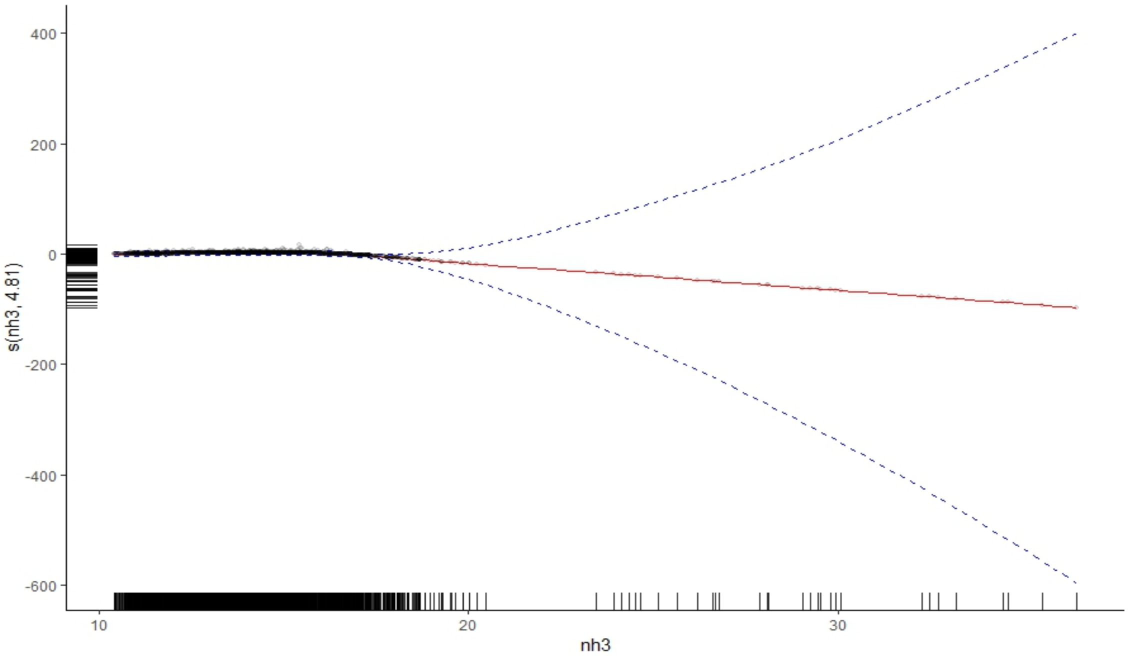

*Adjusted for the lag days, time, maximum temperature, and humidity
